# Transcriptome Analyses Reveal Lipid Metabolic Process in Liver Related to the Difference of Carcass Fat Content in Rainbow Trout (*Oncorhynchus mykiss*)

**DOI:** 10.1155/2016/7281585

**Published:** 2016-08-29

**Authors:** Guo Hu, Wei Gu, Peng Sun, Qingli Bai, Bingqian Wang

**Affiliations:** National and Local Joint Engineering Laboratory for Freshwater Fish Breeding, Heilongjiang River Fishery Research Institute, Chinese Academy of Fishery Sciences, Harbin 150070, China

## Abstract

Excessive accumulation of carcass fat in farm animals, including fish, has a significant impact on meat quality and on the cost of feeding. Similar to farmed animals and humans, the liver can be considered one of the most important organs involved in lipid metabolism in rainbow trout (*Oncorhynchus mykiss*). RNA-seq based whole transcriptome sequencing was performed to liver tissue of rainbow trout with high and low carcass fat content in this study. In total 1,694 differentially expressed transcripts were identified, including many genes involved in lipid metabolism, such as* L-FABP*,* adiponectin*,* PPAR-α, PPAR-β,* and* IGFBP1a*. Evidence presented in this study indicated that lipid metabolic process in liver may be related to the difference of carcass fat content. The relevance of* PPAR-α* and* PPAR-β* as molecular markers for fat storage in liver should be worthy of further investigation.

## 1. Introduction

Excessive fat accumulation in farm animals, including fish, has a significant impact on meat quality and the cost of feeding [1, 2]. The management of fat deposition in farm animals has drawn increasing attention from a growing number of researchers and the public. As we know, body fatness is a heritable, quantitative trait in rainbow trout (*Oncorhynchus mykiss*), and genetic selection has been successfully used to change the muscle fat content in rainbow trout [3, 4]. A good understanding of the physiological mechanisms of fat deposition could be essential to genetic enhancement of the production performance of rainbow trout.

Rainbow trout originated in North America and was introduced to China from the Democratic People's Republic of Korea (North Korea) in 1959 [5, 6]. According to statistical data provided by the Food and Agriculture Organization (FAO), rainbow trout is the major cold-water fish farmed in China, and the production amount was approximately 23,000 tons in 2013 [7]. Additionally, in many research areas, such as immunology and environmental carcinogenesis, this organism has been used as a model animal [8]. The draft genome has been sequenced and several genetic materials and genomics tools have been developed for research [9], facilitating our understanding of the whole genomic physiological mechanisms of the fatness trait in rainbow trout.

Similar to farm animals and humans, lipogenesis, triglyceride accumulation, and metabolic homeostasis occur essentially in the liver of rainbow trout [10]. The liver can be considered one of the most important organs involved in lipid metabolism, and the different expression of functional genes involved in lipid synthesis, degradation, transportation, and storage has been analyzed in the livers of trout strains divergently selected for muscle fat content [11–13]. The research showed that genes involved in lipid metabolism might be influenced by divergent selection and lead to a different model of fat storage in the genetically selected lean and fat trout strains [13]. Another later study indicated that lipid and glucose metabolism was regulated by insulin in two experimental rainbow trout lines [14]. In addition, some evidence has suggested that TOR signaling pathway-associated lipogenesis could be overactivated and the utilization of glucose in the liver might be also improved by the genetic selection [11, 15, 16].

In the present study, RNA-seq based transcriptome sequencing was performed to liver tissue of rainbow trout with extremely high and low carcass fat content. The objectives were to identify the differential expressed transcripts and get meaningful information about the molecular mechanism of fat deposition. The results may be helpful in determining more powerful target genes associated with carcass fat content and would potentially be applied in the development of molecular genetic markers for fatness traits in rainbow trout.

## 2. Materials and Methods

### 2.1. Experimental Animals and Sampling

In this study, a freshwater strain of rainbow trout, selected for large-size body weight and measurement traits using family based BLUP method, was used as experimental material [17]. The breeding program was performed at the Bohai cold-water fish experimental station, located near Jingbo Lake (latitude 44.02°N, longitude 128.74°E), in Heilongjiang Province, China. Water was supplied to the tanks and ponds at the Bohai experimental station using natural flowing spring water (5.2–18.0°C) with a water flow of 20–30 L/sec and a dissolved oxygen concentration ranging from 7 to 11 mg/L. Seventy-five full-sibling families were established using 75 male and 75 female fish for each generation based on passive integrated transponder (PIT) tag. After each family was separately hatched from eggs, the fish absorbed their yolk sacs (30 to 35 days) before swimming up and beginning to feed on a commercial trout fry diet. Each candidate family was then reared separately in a tank (1-meter diameter, 0.5-meter water depth, average water temperature ranging from approximately 6.5°C to 12°C, and water flow at 6 to 8 L/min) for 10 months until reaching a size of >50 g. Fifty fish with no deformities were randomly selected from each tank with PIT tags and were then deposited together in cemented pools (5-meter width, 30-meter length, and 0.7-meter water depth) for one year. The fish beyond two years of age were cultured in earth ponds (15-meter width, 120-meter length, and 0.8-meter water depth). It takes approximately 3–3.5 years to reach sexual maturity for rainbow trout at the Bohai station. All of the fish were fed according to the feeding program for trout from BioMar. All of the animal work was conducted according to the guidelines for the care and use of experimental animals established by the Ministry of Science and Technology of China (document number: 2006-398) and was approved by the Academic Ethics Committee of Heilongjiang River Fishery Research Institute.

Before the traits were measured, the fish were anaesthetized using 0.5 mg/L 2-phenoxyethanol (C_8_H_10_O_2_) to avoid injury from handling. Each fish was measured for carcass fat content using a Fish Fatmeter (Distell Company, Old Levenseat, Scotland, UK) (Model FFM-692; calibration: TROUT-2; representing fat content of whole carcass including belly cavity and fish roe) [18], for body weight using electronic scales (0.1 g), and for total length using Vernier calipers (0.1 cm); each fish was sexed using secondary sexual characteristics, and at the same time, body measurement traits were assessed. All of phenotypic records of carcass fat content related to 1,422 individuals at 2.5 years of age were used for the calculation of the population mean (7.14 ± 1.52%). Six female fish were used for transcriptome sequencing. To ensure that every individual within and between the high and low carcass fat content groups used for RNA sequencing had the same genetic background but no direct kinship, a coancestry analysis was performed based on PIT tag ID of the individuals and the pedigree records. Three female rainbow trout with low carcass fat content (4.20 ± 0.31%) and the same number of high carcass fat content female ones (11.27 ± 0.31%) were randomly selected from the population for transcriptome sequencing. There were extremely significant differences (approximately 3-fold) in carcass fat content (*p* < 0.001) but no significant difference in body weight between the two groups (*p* > 0.05).

### 2.2. Total RNA Extraction, RNA-Seq Library Construction, and Sequencing

The livers of three low carcass fat content female fish and three high carcass fat content female fish were sampled under RNAse-free conditions to perform RNA extraction for sequencing. All of the tissue samples were frozen in liquid nitrogen and stored at −80°C until analysis. Total RNA was isolated using Trizol reagent (Invitrogen, Carlsbad, CA, USA), and RNA degradation and contamination were monitored on 1% agarose gels. Then, the clustering and sequencing were performed by the Experimental Department of Novogene Ltd. The clustering of the index-coded samples was performed on a cBot Cluster Generation System using a TruSeq SR Cluster Kit v3-cBot-HS (Illumina), according to the manufacturers' instructions. After cluster generation, the library preparations were sequenced on an Illumina HiSeq 2500 platform, and 125 bp paired-end reads were generated.

### 2.3. RNA-Seq Data Processing, Annotation, and Differential Expression Identification

Raw counts of RNA-seq reads for each transcript and in each sample were derived and normalized to fragments per kilobase of exon per million fragments mapped reads (FPKM). All transcriptome data from six individuals were aligned to the* Oncorhynchus mykiss* mRNA dataset (*Oncorhynchus mykiss* genome project accession via http://www.genoscope.cns.fr/trout/) using the TopHat [19], and the expression of trout transcripts was evaluated using the software Cufflinks [20]; TopHat and Cufflinks were performed with default parameters. Differentially expressed transcripts (fold changes ≥2 or fold changes ≤0.5 and adjusted* p* value ≤0.01) between high and low carcass fat content fish were identified with the Cufflink package based on FPKM; visualization of transcripts expression was performed using R software [21]. GO terms were assigned to the transcripts according to their corresponding homologs in the trout mRNA database (http://www.genoscope.cns.fr/trout/). The GO annotation result was visualized in WEGO [22]. Lastly, the GO terms enrichment analysis for the differentially expressed transcripts was carried out by topGO package [23].

### 2.4. Validation of Differentially Expressed Genes by Real-Time Quantitative RT-PCR

Fourteen differentially expressed genes identified by the transcriptome sequencing were validated by real-time quantitative RT-PCR, using the same fish sample, and EF1*α* was used as a reference control [11]. Real-time RT-PCR was performed using SYBR (R) GREEN I NUCLEIC A (Life Technologies) on the LightCycler 480II Real-Time System (Roche, Switzerland). The reaction was performed using the following conditions: denaturation at 95°C for 3 min, followed by 40 cycles of amplification (95°C for 30 s, 60°C for 30 s, and 60°C for 45 s). Relative expression was calculated using the delta-delta-Ct method; primer sequences can be found in Table 1.

## 3. Results

### 3.1. Sequencing of Liver Transcriptome in Rainbow Trout

The liver transcriptome expression of six fish at 2.5 years was analyzed by RNA sequencing. After removing low-quality reads, adaptor, and barcode sequences, nearly 35 million clean reads for each individual were obtained; the descriptive statistics of the RNA-seq reads for each individual are shown in Table 2. All of the raw data were submitted to the NCBI database (accession numbers SRX1067664 and SRR2072562).

### 3.2. Gene Functional Annotation for the Differentially Expressed Transcripts

**Figure 1:**
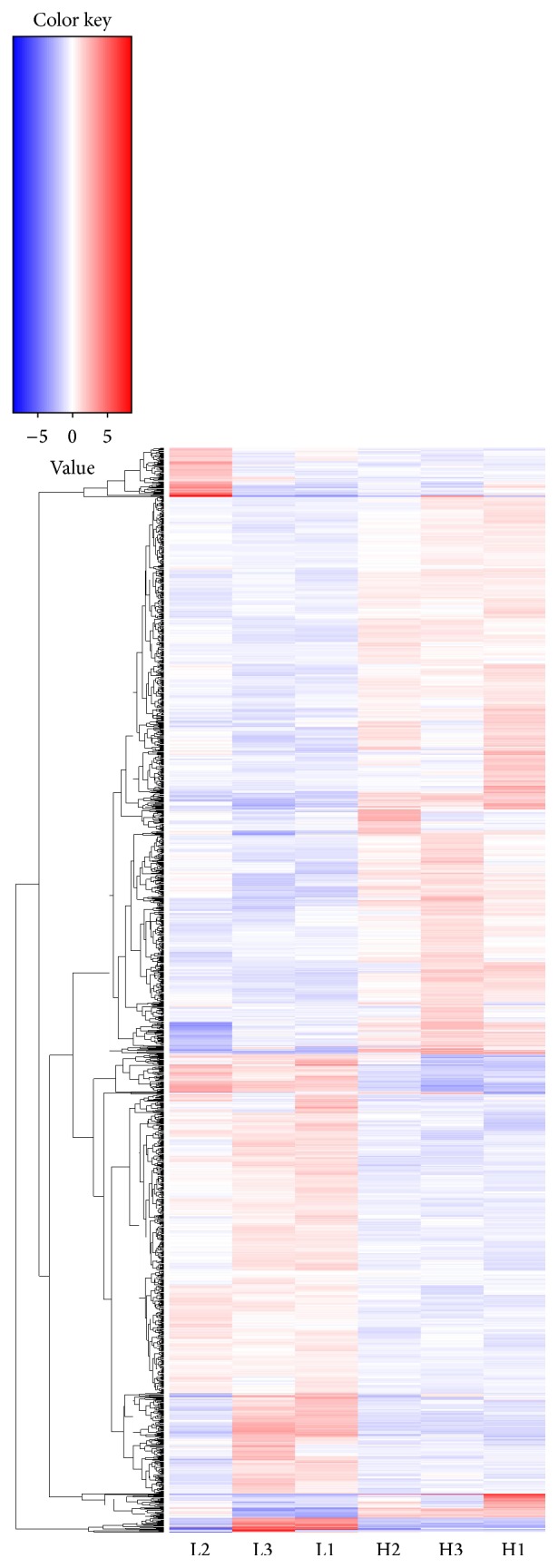
Cluster image of 1,694 significant differentially expressed transcripts between the high and low carcass fat content rainbow trout. A total of 1,694 differentially expressed transcripts were identified with Cufflink package based on FPKM: 912 were highly expressed in rainbow trout with high carcass fat content, and 782 showed higher expression level in those fish with low carcass fat content. H1, H2, and H3 represent the first, second, and third fish in the high carcass fat content group, respectively; and L1, L2, and L3 represent the first, second, and third fish in the low carcass fat content group, respectively. Colored bars indicate relative expression levels. Transcripts expressed at higher levels were assigned red, while transcripts expressed at low levels were assigned blue.

A total of 1,694 differentially expressed transcripts were identified from liver tissue between the high and low carcass fat content rainbow trout (Figure 1). The standard nomenclature, expression, and gene annotation for the differentially expressed transcripts were shown in Table S1 in Supplementary Material available online at http://dx.doi.org/10.1155/2016/7281585. Among these transcripts, 912 transcripts were highly expressed in high carcass fat content fish, including many key factors of lipogenesis and fat deposition in rainbow trout, such as peroxisome proliferator activated receptor alpha (*PPAR-α*),* PPAR-β*, insulin-like growth factor binding protein 1a (*IGFBP1a*), and fatty acid-binding protein 10-A, liver (*L-FABP*). At the same time, 782 transcripts showed higher expression levels in low carcass fat content fish, including many transcription factors with important effects on growth, differentiation, lipid metabolism, carbohydrate metabolism, and immunity, for example, growth hormone receptor isoform 1 (*GHR1*), insulin-induced gene 1 (*INSIG1*), and heat shock 70 kDa protein (*HSP70*).

**Figure 2:**
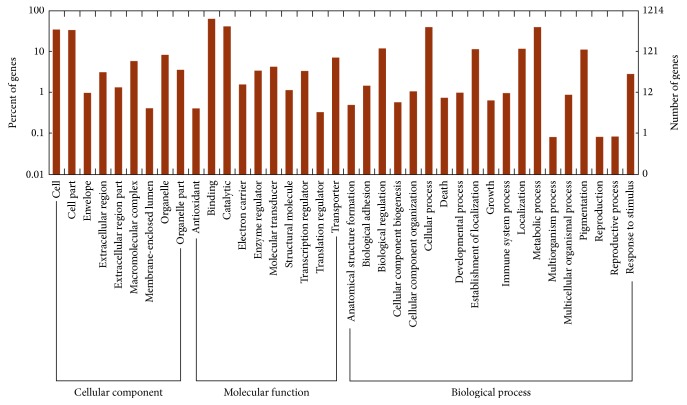
GO annotation results of the differently expressed transcripts between the high and low carcass fat content rainbow trout.

GO annotations provided in the trout mRNA database (*Oncorhynchus mykiss *genome project accession via http://www.genoscope.cns.fr/trout/) were used in this study. At least one in three main categories of GO terms (biological process, molecular function, and cellular component) was assigned to all 1,694 differentially expressed transcripts and then was classified into different functional categories, as shown in Figure 2. The results showed that metabolic process (GO: 0008152) was the most abundant group in the biological process category, followed by cellular process (GO: 0009987), while biological regulation (GO: 0065007) and establishment of localization (GO: 0051234) were also common, consistent with the central role of liver in the basal and intermediary metabolism. In the molecular function category, the most abundant group was binding (GO: 0005488), and the second most abundant one was catalytic activity (GO: 0003824). There were also transcripts classified into specific groups, such as transporter activity (GO: 0005215), molecular transducer activity (GO: 0060089), enzyme regulator activity (GO: 0030234), and transcription regulator activity (GO: 0030528), indicating that many genes were involved in important physiological functions of synthesis, transport, and catabolism in the livers of rainbow trout. In the cellular component category, the most abundant groups were related to cell (GO: 0005623), cell part (GO: 0044464), organelle (GO: 0043226), organelle part (GO: 0044422), and macromolecular complex (GO: 0032991).

### 3.3. GO Enrichment Analysis for Differentially Expressed Transcripts

The results of GO enrichment analysis for 1,694 differentially expressed transcripts were shown in Table S2 in the Supplementary Material. GO terms in the biological process category were highly enriched, with 84 GO terms attaining significant levels, including GO: 0044710 (single-organism metabolic process), GO: 0055114 (oxidation-reduction process), GO: 0006520 (cellular amino acid metabolic process), GO: 0006629 (lipid metabolic process), GO: 0006006 (glucose metabolic process), GO: 0019318 (hexose metabolic process), GO: 0005996 (monosaccharide metabolic process), and GO: 0006541 (glutamine metabolic process), consistent with the physiological functions of the livers of rainbow trout. At the same time, the GO terms in the molecular function category were also highly enriched, and 88 GO terms reached the significant level, including GO: 0016491 (oxidoreductase activity), GO: 0016298 (lipase activity), GO: 0048037 (cofactor binding), GO: 0050662 (coenzyme binding), GO: 0005319 (lipid transporter activity), and GO: 0004465 (lipoprotein lipase activity), indicating that the lipid metabolism mode might be different between high and low carcass fat content fish.

**Figure 3:**
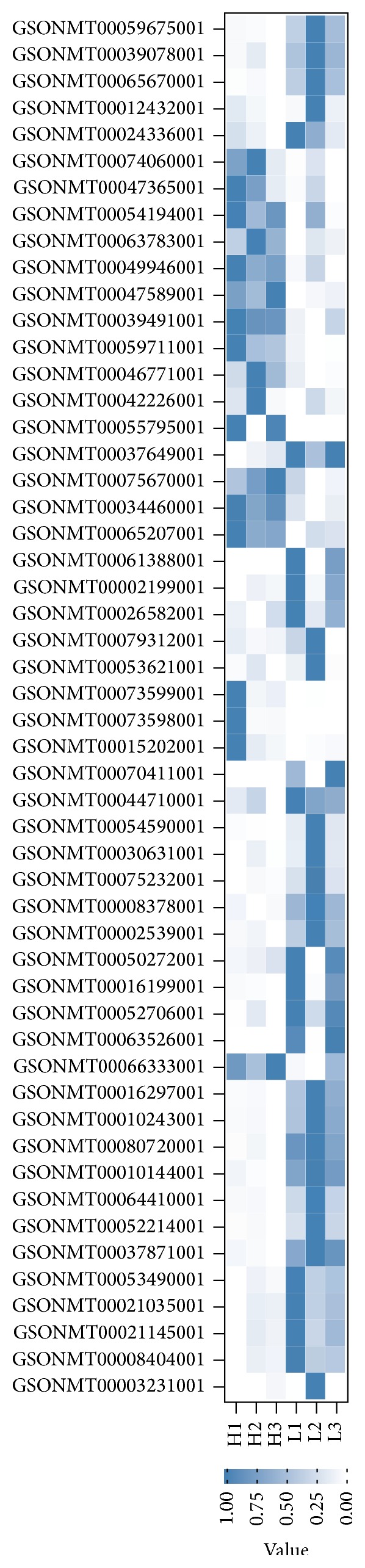
Heat map showing the expression profiles of differentially significant transcripts involved in lipid metabolic processes. A total of 52 of 372 transcripts involved in lipid metabolic processes (GO: 0006629) were differentially expressed between the high and low carcass fat content fish groups. H1, H2, and H3 represent the first, second, and third fish in the high carcass fat content group, respectively; L1, L2, and L3 represent the first, second, and third fish in the low carcass fat content group, respectively. Colored bars indicate relative expression levels. Transcripts expressed at higher levels were assigned blue, while transcripts expressed at low levels were assigned white.

Moreover, we analyzed the expression of transcripts grouped as “lipid metabolic process” GO term (0006629) based on the transcriptome data, revealing that 52 of 372 transcripts (14%) were differentially expressed between the high and low carcass fat content fish groups (Figure 3), providing meaningful information about the difference in lipid metabolism between the two groups. The standard nomenclature, expression, and gene annotation for the 52 transcripts were shown in Table S3.

### 3.4. Validation of Differentially Expressed Genes by Real-Time Quantitative RT-PCR

To validate the results of differentially expressed genes in transcriptome sequencing, we performed real-time quantitative RT-PCR for 14 differentially expressed genes involved in lipid metabolism, growth regulation, and other important functions. Among these genes,* L-FABP*, putative* I-FABP*,* IGFBP1a*, insulin-like growth factor binding protein, acid labile subunit (*IGFBP-ALS*),* adiponectin, cytochrome P450, PPAR-α, PPAR-β*, and lipopolysaccharide-binding protein (*LBP*) were highly expressed in the high carcass fat content fish group, while heat shock 70 kDa protein (*HSP70*), acetyl-CoA acetyltransferase, cytosolic (*ACAT2*), StAR-related lipid transfer protein (*SATRT*),* GHR1*, and* INSIG1* showed lower expression level in the high carcass fat content fish group. The results of differentially expressed genes identified by real-time quantitative RT-PCR had good consistency with the transcriptome data. For transcriptome data, the evaluation criteria for differentially expressed genes between the high and low carcass fat content fish groups were fold changes ≥2 or fold changes ≤0.5 and adjusted *p* value ≤ 0.01, while for real-time quantitative RT-PCR we used Student's* t*-test (*p* < 0.05). All fourteen genes detected by real-time RT-PCR reached significance. The fold change results of RNA sequencing and real-time RT-PCR for 14 differentially expressed genes were compared in Table 3.

## 4. Discussion

As is well known, the Chinese believe that the best tasting and most nutritious aquatic animals must be alive immediately prior to cooking, and they eat whole fish from beginning to end (with head and tail); thus, the fat content of the whole carcass of the fish body is an economically important trait in China. Fatty acid de novo synthesis in rainbow trout occurs mainly in the liver [10, 24], and several studies have explored the correlations between gene expression in liver tissues and lipid metabolism [25–27].

### 4.1. Differentially Expressed Functional Genes Involved in Lipid Metabolism and Energy Balance

Among the 1,694 differentially expressed genes,* L-FABP* was detected to be more than 30-fold higher expressed in the fat group than in the lean group by both transcriptome sequencing and real-time RT-PCR methods. Liver type fatty acid-binding protein is a functional protein with a small molecular weight that belongs to a superfamily of lipid-binding proteins and participates in intracellular fatty acid transportation in human, farm animals, birds, and fish [28–30]. Higher expression of the* L-FABP* gene in the livers of the high carcass fat content group than in the low carcass fat content group might be the result of increased fatty acid transportation into white muscle, skin, and other parts, leading to more fat deposition in the whole carcass. The results indicated that the expression of the* L-FABP* gene should have a positive effect on fat deposition in the rainbow trout.

The PPAR gene superfamily belongs to the nuclear receptor superfamily, including three different genes—*PPAR-α, PPAR-β, *and* PPAR-γ. PPAR*s are a type of ligand-activated transcription factors that control gene expression by binding to specific response elements (PPREs) within promoters [31].* PPAR*s play critical physiological roles as lipid sensors and regulators of lipid metabolism and can transactivate multiple target genes and interact with other transcription factors involved in lipid metabolic pathways [32, 33]. In the current study,* PPAR-α* and* PPAR-β* were expressed significantly more in the high carcass fat content fish group than in the low carcass fat content fish group by transcriptome sequencing and as validated by real-time RT-PCR. Our results regarding* PPAR-α* expression were different from Kolditz et al.'s results on the divergent selection of lean and fat fish for muscle fat content by cDNA microarray in rainbow trout [13]. In their work,* PPAR-α* expression was lower in the fat line than in the lean line. The differences between our results and the result of Kolditz et al. might have been caused in part by different ages of the fish and varying aquaculture environmental conditions. Other potential reasons for the difference in PPAR-*α* expression between the high and low fat content fish in the two populations included the different histories of the two populations. In addition, in contrast to* PPAR-α* and* PPAR-β*, the expression of* PPAR-γ* in the liver was very low and was not differentially expressed between the fat and lean group, consistent with the expression level of* PPAR-γ* gene in liver tissue being only 10% to 30% of the level in adipose tissue in mammals [34, 35] and similar to that in the chicken [36]. In the current study, the gene expression of* PPAR-α* and* PPAR-β* was highly correlated with fat deposition, suggesting that they were also key factors of fat accumulation in the rainbow trout carcass.

Cytochrome P-450 is transcriptionally modulated by glucocorticoid binding to glucocorticoid response elements in the promoter region, playing important roles in the oxidative metabolism of endogenous and exogenous compounds [37].* Cytochrome P-450* was detected to be more than 30-fold higher expressed in the fat group than in the lean group by transcriptome sequencing, and it was validated to be nearly 20-fold higher by real-time RT-PCR. This result was in agreement with the currently known oxidation-reduction process of gene expression in the liver. The insulin regulated lipid metabolism of fish does not seem to follow mammalian patterns; the insulin-like growth factors (IGFs) are polypeptides with high sequence similarity to insulin, and they are able to modulate lipolysis and lipogenesis in fish hepatocytes [14–16, 38]. Insulin-like growth factor binding proteins (IGFBPs) regulate the biological functions of IGFs, and IGFs can stimulate the growth of multiple tissue cell types [39]. In the current study, the gene expression of two* IGFBP* isoforms and gene expression of* INSIG1* were differently expressed between the lean and fat fish groups, suggesting potential roles of insulin and insulin-like growth factors in lipid storage or utilization of fat accumulation in rainbow trout.

In total, many functional genes involved in lipid and carbohydrate metabolism might contribute to phenotypic variations in carcass fat content, and they were found to be expressed in both high and low carcass fat content fish groups. However, some of these genes were expressed significantly different between these two groups. On the one hand, this phenomenon could partly occur because of potential positive genetic correlation between growth traits and carcass fat content and the growth traits being under strong artificial selection in this experimental population. On the other hand, there was a large difference in the group mean of carcass fat content between the lean and fat groups, suggesting that the dynamics of genetics effects and the gene expression mode might be influenced by genetic heterogeneity between the lean and fat groups.

### 4.2. Metabolic Process Related to the Difference of Carcass Fat Content Based on GO Enrichment Analysis

The GO approach could help to predict the functions of genes based on the existing architecture and prior knowledge of molecular biological mechanisms for nonmodel organisms. In the present study, a large number of functional genes were significantly enriched in lipid and carbohydrate metabolism related GO terms, such as GO: 0006629 (lipid metabolic process), GO: 0006006 (glucose metabolic process), GO: 0019318 (hexose metabolic process), and GO: 0005996 (monosaccharide metabolic process), which was consistent with liver being an important tissue in the biological process of lipid and carbohydrate metabolism and metabolic energy balance. In detail, we identified 52 differentially expressed transcripts significantly enriched in the lipid metabolic process (GO: 0006629) and 20 differentially expressed transcripts significantly enriched in the glucose metabolic process (GO: 0006006). In farm animals, fatty acids and glucose are the two main metabolic substrates oxidized by animals for energy production. The mobilization of fat stores could be decreased by glucose, using both inhibiting lipolysis and stimulating primary reesterification approaches. Our results were consistent with this information and also suggested that the expression and regulation of lipid and carbohydrate metabolism genes should play key roles in the ontogenesis of carcass fat content in rainbow trout.

### 4.3. Consistency between Transcriptome Sequencing and Real-Time RT-PCR

As is known, transcriptome sequencing and real-time RT-PCR analysis are both powerful tools for obtaining a view of gene expression. However, quantifying gene expression by sequencing and real-time RT-PCR is not necessarily consistent. Although much meaningful information can be obtained from transcriptome sequencing alone, this technology still has limitations in accuracy and in fairness for RNA-seq data processing [40]. Although fold changes were different for some genes between these two methods, all fourteen of the genes detected by real-time RT-PCR attained significant levels of differential expression (*p* < 0.05), conferring robustness to our transcriptome dataset.

## 5. Conclusion

In this study, the transcript expression profile of the liver tissue in rainbow trout was investigated comprehensively using RNA sequencing and was confirmed by real-time RT-PCR. A total of 1,694 transcripts were differentially expressed between high and low carcass content fish group. Evidence presented in this study indicated that lipid metabolic process in liver may be related to the difference of carcass fat content. The relevance of* PPAR-α* and* PPAR-β* as molecular markers for fat storage in liver commands further investigation.

## Supplementary Material

The detailed information about the 1,694 differentially expressed transcripts identified from liver tissue between the high and low carcass fat content fish were shown in the Supplementary Materials, including standard nomenclature, expression and gene annotation provided by the Trout Genome dataset and the GO enrichment results by using the topGO package.

## Figures and Tables

**Table 1:** The information of the primer pairs used to analyze gene expression by real-time quantitative RT-PCR.

Standard nomenclature^*∗*^	Primer	Product size (bp)
GSONMT00021034001	5′ CTGGGCACCACCTACTCAT 3′	110
	5′ GTGTCTGTGAAGGCAACG 3′	
GSONMT00026574001	5′ AAACTGCTGGTGAAGACG 3′	136
	5′ TGTTGAGGACAAAGAGGG 3′	
GSONMT00028411001	5′ CCCTGGGCGTACAGTTTGAC 3′	188
	5′ CTTGGCATCCACTCCATCGT 3′	
GSONMT00029574001	5′ TTCAAGGGTCGTGGGAGA 3′	159
	5′ CGGAGGGTGTTGGAAGTG 3′	
GSONMT00032762001	5′ AGCAGAACGGCAATGACT 3′	181
	5′ TCCTGGACGCTGGAGAAT 3′	
GSONMT00051137001	5′ GGAGCCTGGTTGTGGATG 3′	286
	5′ GCTGTGGCCGTGGAGATA 3′	
GSONMT00051158001	5′ CCTGGCTCTGTTTGTGGC 3′	137
	5′ TGTCGTCTGGGTGGTTGG 3′	
GSONMT00054447001	5′ CTGGAGTAAAGATGGGTGAC 3′	169
	5′ GTCCTGTTCTGGGATTGG 3′	
GSONMT00060652001	5′ CCGACCACCAACCCTAAT 3′	272
	5′ CACTGGCAGCGGTAGAAC 3′	
GSONMT00062349001	5′ GCCTGGATGAGAATGA 3′	335
	5′ GATACCGCAGGACAAT 3′	
GSONMT00064682001	5′ CCTACTGAGCCCATTCCT 3′	360
	5′ ATGGAGACTAAGCGAGGC 3′	
GSONMT00070501001	5′ CGCCCACCTCTAAACAAGCC 3′	459
	5′ GCAGCGTCATCCAGCCCATC 3′	
GSONMT00077785001	5′ CGTGCCCATCCGTTTCAATA 3′	137
	5′ CCCGAGCATCTTTGGTGTAG 3′	
GSONMT00082167001	5′ ACCGTGGGAGTAGTTCTTGC 3′	425
	5′ TAGACACCGTTGTAGACCAG 3′	
EF1*α*	5′ TCCTCTTGGTCGTTTCGCTG 3′	159
	5′ ACCCGAGGGACATCCTGTG 3′	

^*∗*^Note: the standard nomenclature for rainbow trout according to GBrowse-2.54 of rainbow trout genome project database.

**Table 2:** The descriptive statistics of the raw RNA-seq datasets for all of the six individuals sequenced in the present study.

Sample	Raw reads	Clean reads	Clean bases	Error (%)	Q20 (%)	Q30 (%)	GC (%)
H1_1	17429887	16907974	2.11G	0.03	96.27	92.45	50.25
H1_2	17429887	16907974	2.11G	0.04	93.31	87.75	50.2
H2_1	18051923	17574994	2.2G	0.03	96.18	92.3	50.37
H2_2	18051923	17574994	2.2G	0.04	93.56	88.15	50.33
H3_1	16777106	16241788	2.03G	0.03	96.16	92.23	50.07
H3_2	16777106	16241788	2.03G	0.04	93.33	87.71	50.04
L1_1	18535584	17957775	2.24G	0.03	96.41	92.72	49.7
L1_2	18535584	17957775	2.24G	0.04	93.97	88.84	49.66
L2_1	18083733	17537741	2.19G	0.03	96.26	92.43	49.72
L2_2	18083733	17537741	2.19G	0.04	94.03	88.89	49.68
L3_1	19420929	18772655	2.35G	0.03	96.24	92.41	50.08
L3_2	19420929	18772655	2.35G	0.04	93.66	88.3	50.05

Notes: sample name: H1 is the first fish in high carcass fat content group and so on; L1 is the first fish in high carcass fat content group and so on; H1_1 is the left-end reads, H1_2 is the right-end reads; the descriptive statistics for the individual H1 is the sum of H1_1 and H1_2 and so on.

**Table 3:** Comparison of fold differences in 14 differentially expressed genes detected by transcriptome sequencing and real-time RT-PCR analyses.

Standard nomenclature^*∗*^	Fold change by real-time RT-PCR	Fold change by RNA-seq	Gene annotation
GSONMT00021034001	0.25 ± 0.03	0.18	Heat shock 70 kDa protein
GSONMT00026574001	1.40 ± 0.12	2.70	Lipopolysaccharide-binding protein/bactericidal permeability-increasing protein
GSONMT00028411001	4.84 ± 0.35	4.44	Fatty acid-binding protein, intestinal putative mRNA
GSONMT00029574001	36.83 ± 2.30	17.70	Cytochrome P450
GSONMT00032762001	33.50 ± 1.95	35.73	Fatty acid-binding protein 10-A, liver
GSONMT00051137001	6.05 ± 0.50	4.27	Insulin-like growth factor binding protein 1a
GSONMT00051158001	1.50 ± 0.15	3.03	Peroxisome proliferator activated receptor alpha
GSONMT00054447001	0.18 ± 0.04	0.18	Acetyl-CoA acetyltransferase, cytosolic
GSONMT00060652001	2.22 ± 0.15	2.66	Peroxisome proliferator activated receptor beta
GSONMT00062349001	0.13 ± 0.02	0.19	StAR-related lipid transfer protein
GSONMT00064682001	2.80 ± 0.15	2.76	Insulin-like growth factor binding protein, acid labile subunit
GSONMT00070501001	0.26 ± 0.03	0.11	Growth hormone receptor isoform 1
GSONMT00077785001	3.22 ± 0.19	17.14	Adiponectin
GSONMT00082167001	0.71 ± 0.05	0.29	Insulin-induced gene 1

^*∗*^Note: the standard nomenclature for rainbow trout according to GBrowse-2.54 of rainbow trout genome project database.
