# Supplementary figures and images for: Transcriptome Analyses Reveal Lipid Metabolic Process in Liver Related to the Difference of Carcass Fat Content in Rainbow Trout (Oncorhynchus mykiss)
Source: Int J Genomics. 2016 Aug 29;2016:7281585. doi: 10.1155/2016/7281585 (PMC5019904; doi:10.1155/2016/7281585)

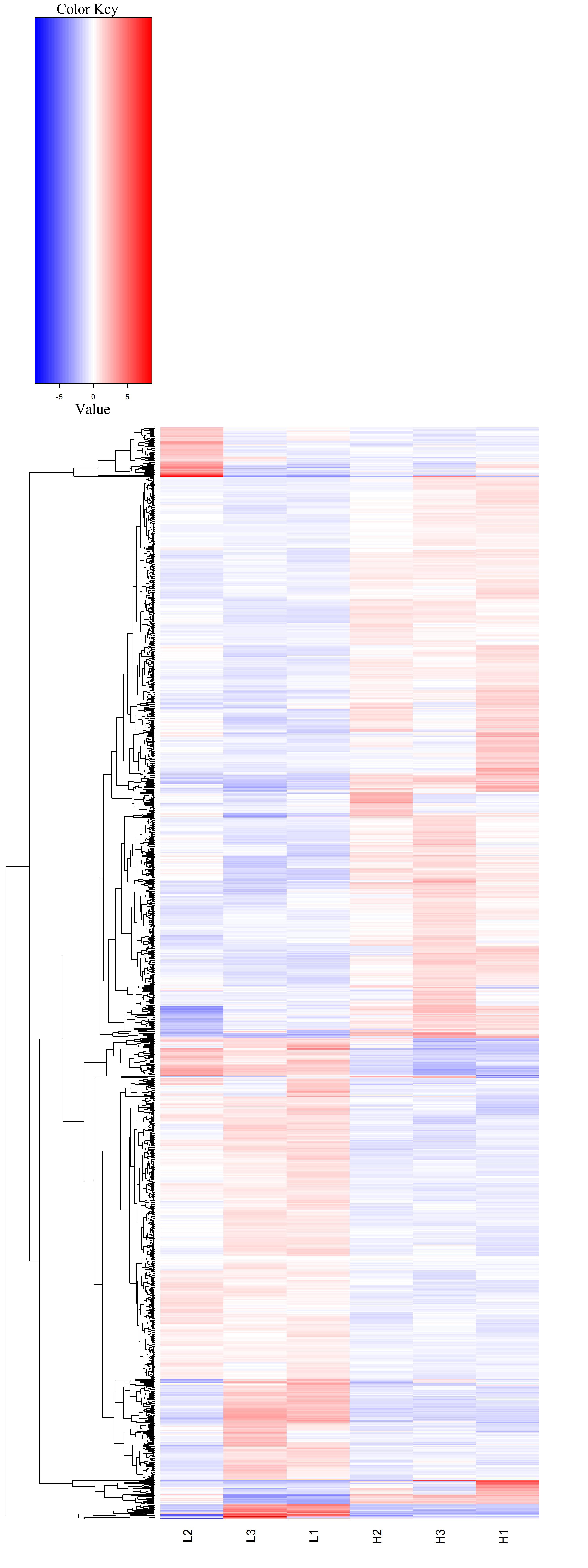

Supplement: Supplementary file 1 — The detailed information about the 1,694 differentially expressed transcripts identified from liver tissue between the high and low carcass fat content fish were shown in the Supplementary Materials, including standard nomenclature, expression and gene annotation provided by the Trout Genome dataset and the GO enrichment results by using the topGO package. [file 7281585.f1.zip › Figures_and_Supplementary Tables/Figures/Figure1_revised.jpg]

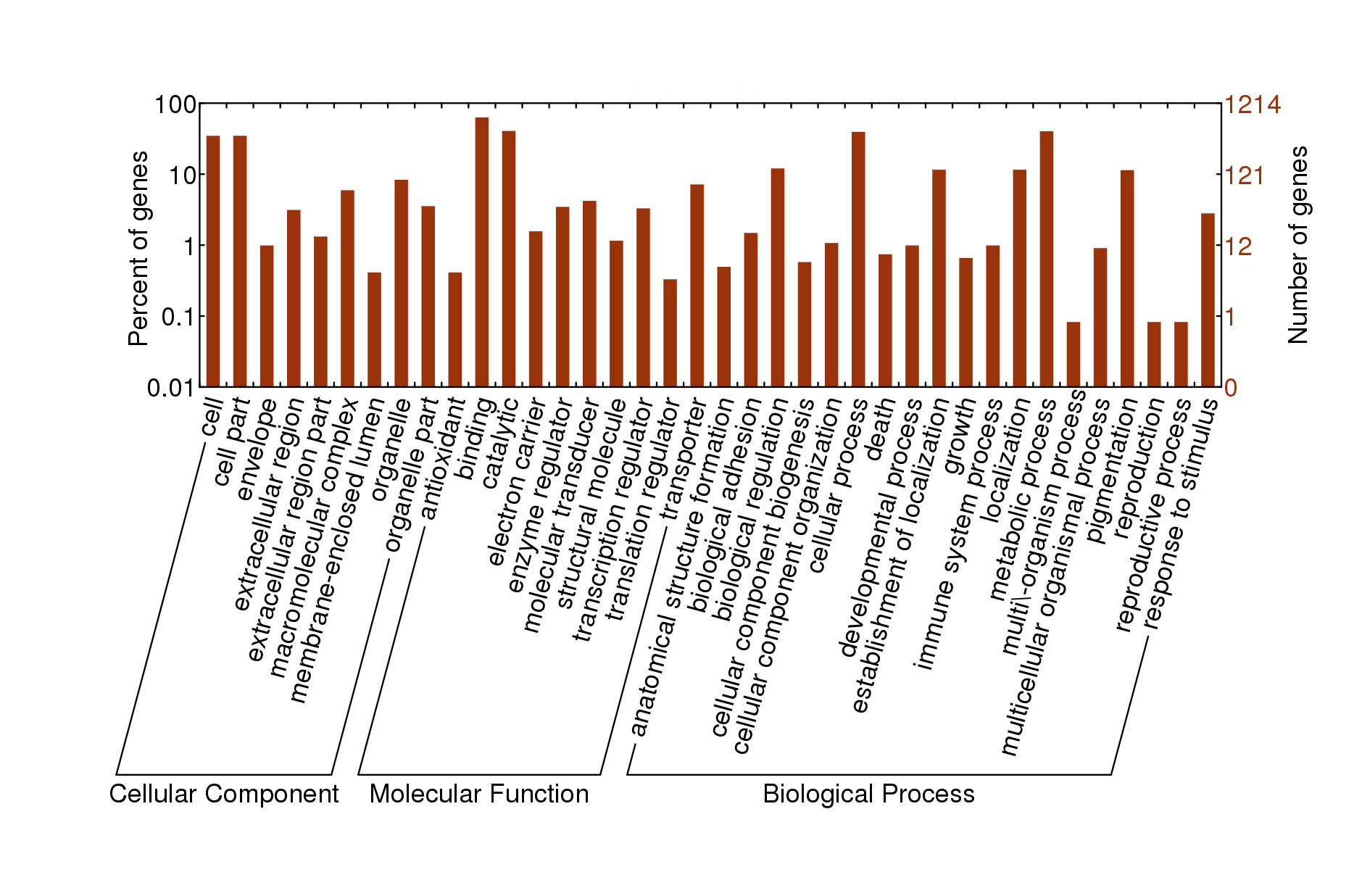

Supplement: Supplementary file 1 — The detailed information about the 1,694 differentially expressed transcripts identified from liver tissue between the high and low carcass fat content fish were shown in the Supplementary Materials, including standard nomenclature, expression and gene annotation provided by the Trout Genome dataset and the GO enrichment results by using the topGO package. [file 7281585.f1.zip › Figures_and_Supplementary Tables/Figures/Figure2.jpg]

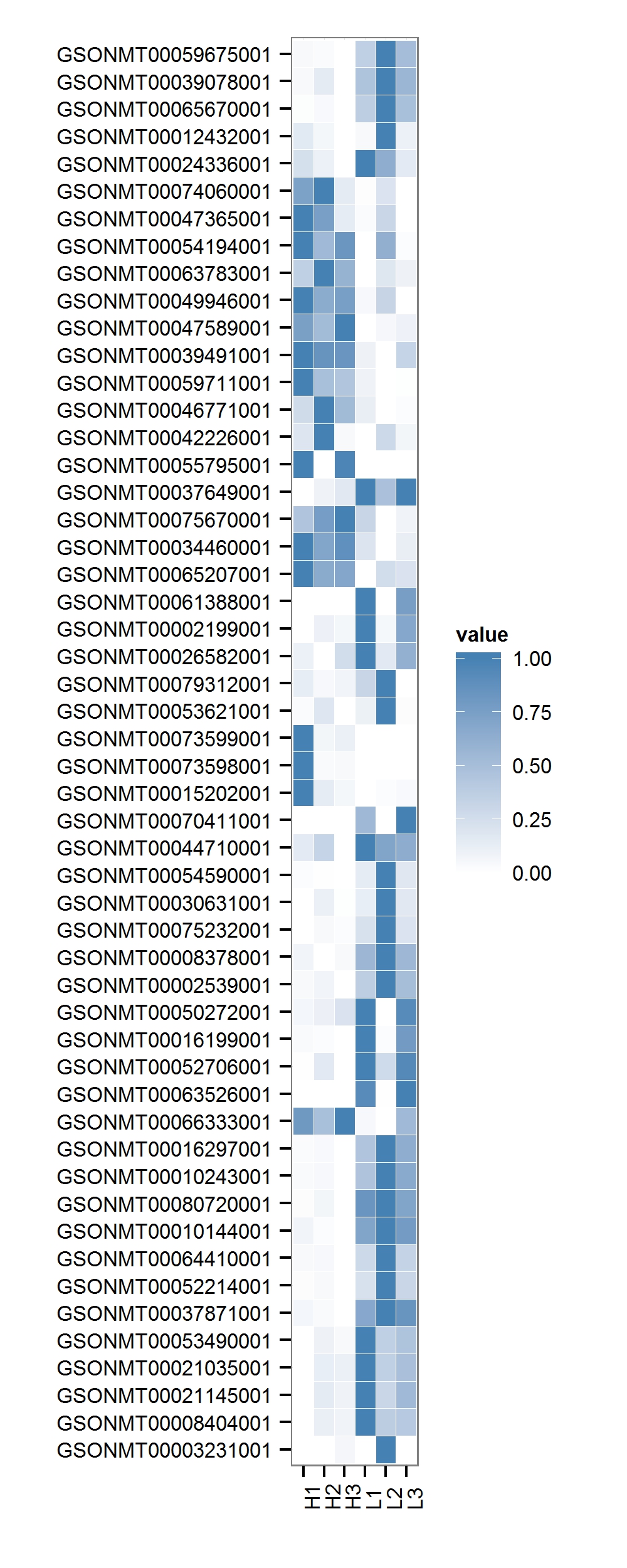

Supplement: Supplementary file 1 — The detailed information about the 1,694 differentially expressed transcripts identified from liver tissue between the high and low carcass fat content fish were shown in the Supplementary Materials, including standard nomenclature, expression and gene annotation provided by the Trout Genome dataset and the GO enrichment results by using the topGO package. [file 7281585.f1.zip › Figures_and_Supplementary Tables/Figures/Figure3.jpg]
